# Prototype of Self-Service Electronic Stethoscope to Be Used by Patients During Online Medical Consultations

**DOI:** 10.3390/s25010226

**Published:** 2025-01-03

**Authors:** Iwona Chuchnowska, Katarzyna Białas

**Affiliations:** 1 Faculty of Biomedical Engineering, Silesian University of Technology, Roosevelta 40, 41-800 Zabrze, Poland; 2 Faculty of Mechanical Engineering, Silesian University of Technology, Konarskiego 18A, 44-100 Gliwice, Poland; katarzyna.bialas@polsl.pl

**Keywords:** electronic stethoscope, 3D printing, recognition, detection

## Abstract

This article presents the authors’ design of an electronic stethoscope intended for use during online medical consultations for patient auscultation. The goal of the project was to design an instrument that is durable, user-friendly, and affordable. Existing electronic components were used to create the device and a traditional single-sided chest piece. Three-dimensional printing technology was employed to manufacture the prototype. Following the selection of the material, a static tensile strength test was conducted on the printed samples as part of the pre-implementation investigations. Results: Tests on samples made of PLA with a 50% hexagonal infill demonstrated a tensile strength of 36 MPa and an elongation of 4–5%, which was deemed satisfactory for the intended application in the stethoscope’s manufacture. The designed and manufactured electronic stethoscope presented in the article can be connected to headphones or speakers, enabling remote medical consultation. According to the opinion of doctors who tested it, it provides the appropriate sound quality for auscultation. This stethoscope facilitates the rapid detection and recognition of cardiac and respiratory activity in humans.

## 1. Introduction

In lung diseases and cardiological conditions, the stethoscope is defined as ‘an instrument allowing the listening to the sound/noise emitted by the body’. It has been an indispensable diagnostic instrument for over two hundred years. Its contemporary form was patented in the 1960s [1] and its less complicated predecessor in 1910 [2]. These facts do not exclude the possibility of further search for more advanced and functional versions of the stethoscope. Modifications relate not only to the details of individual parts of the instrument but also to the system of operation and sound transmission. Apart from traditional mechanical stethoscopes based on the transmission of analogue sound (in the form of acoustic waves), new types of stethoscopes are designed by using digital sound transmission [3,4,5]. The conversion of sound into a digital form enables the computer-aided analysis and processing of data in certain software programmes, as well as their recording and transmission.

At the beginning of the year 2020, the necessity of isolation caused by the SARS-CoV-2 pandemic re-organized many areas of life and released creative potential to improve remote forms of contact on different planes. Many companies were forced to shift into remote working mode, which also partly affected the occupational group of physicians. Standard medical check-ups that did not require very urgent direct contact with patients adopted the form of medical online consultation. This fact, however, limited to some extent the possibility of a full examination of the patient [6,7,8].

Medical examinations which previously required direct contact between the patient and the doctor include standard auscultation using the stethoscope in the cases of suspected lung or heart diseases (especially lung conditions require cyclic auscultation). More often than not, in paediatrics, particularly in the initial phase of diseases, pathological changes are impossible to hear; however, they require frequent examination and permanent observation [9]. The development of contemporary technologies can lead to the reduction in direct contact between the physician and patient down to a necessary minimum [10].

### 1.1. Project Objectives

The project aimed to create an auscultation device for the patient’s self-use during a medical consultation online. The adopted assumptions prioritized the following aspects: user-friendliness and affordability resulting from low production costs. To some extent, the designed stethoscope makes use of the components of classical stethoscopes [11].

### 1.2. Structure of Traditional Stethoscopes

A traditional stethoscope consists of two basic parts, i.e., the chest piece (the head) equipped with a diaphragm, whose task is to receive and transmit acoustic vibration from the examined body area, and the headset with tubing transmitting acoustic waves to the surface area of the tympanic membrane (eardrum) of the examining person’s ear.

The chest piece is usually made of metal–stainless steel, chromium-plated (covered by a galvanic coating) brass, or aluminium subjected to electrochemical treatment. The diaphragm (membrane) spread on the chest piece is responsible for the stethoscope’s sensitivity. It is usually manufactured from plastics (e.g., epoxy foil reinforced with glass fibre); however, there are also foils combining plastics with a metal foil made from bronze alloys. Dual-sided chest pieces enjoy great popularity. They are equipped with a dual-position rotation pin. They receive sounds through a diaphragm or a bell located on the opposite side. Nevertheless, single-sided stethoscopes are also often in use and are usually called anaesthesiological stethoscopes. They have a considerably lower chest piece, which facilitates the placing of the instrument under the patient’s clothes.

The second part of the classical stethoscope includes the headset with tubing playing a function of a waveguide guiding acoustic signals from the chest piece to the tympanic membrane of the examining person. The tubing is made of elastic plastics. Stiff end pieces (ear tubes) supported by binaural springs eliminate the necessity of holding up the ends of the tubing (ear pieces) in the ears and guarantees the leak tightness of the acoustic duct. Similar to the chest piece, the headset is made of stainless steel, chromium-plated brass, or aluminium. Essential elements of the stethoscope are the ear pieces, which have an impact on the minimization of noises caused by external background noise. Ear pieces or ear tips are usually made in the form of soft elements self-adjusting to the shape of the ear. They are made of elastic plastics (e.g., nitrile rubber), which prevents the loss of the acoustic signal [12].

### 1.3. Advantages and Disadvantages of Traditional Stethoscopes

Table 1 presents the advantages and disadvantages of using a traditional stethoscope, highlighting its strengths and limitations in medical practice [10,13].

Given the disadvantages, particularly the lack of remote capability, it is worth considering the design of an electronic stethoscope.

## 2. Materials and Methods

The project aimed to create an auscultation device for the patient’s self-use during a medical consultation online. The adopted assumptions prioritized the following aspects: user-friendliness and affordability resulting from low production costs. To some extent, the designed stethoscope makes use of the components of classical stethoscopes.

### 2.1. Authors’ Design of Stethoscope

The presented design of the electronic stethoscope features a classical single-sided chest piece, which is built in the designed main body of the device. The single-sided chest piece was chosen due to the fact that it provides patients with easy self-service and an affordable price. In addition, the chest piece of the stethoscope was incorporated directly into the body of the designed device.

The electronic system, whose components are mounted inside the device body, is responsible for sound transmission. The system consists of components which are widely available on the market. It features a microcontroller, microphone, charging module, battery (lithium-ion cell), micro-USB port, signal diode, switch, and jack socket enabling the connection of headphones or loudspeakers. Among the various available components, the ESP32 microcontroller with a dedicated converter from Adafruit, a company based in the United States, was used.

The selection of this type was dictated by its relatively low power consumption. As for the microphone, the SPH0645 model, manufactured by Adafruit from the United States, operating with the I^2^S bus (Inter-IC Sound Bus) was used as a medium connecting the microcontroller to the audio decoder [14]. The device is powered by a battery (lithium-ion cell 18650 with a capacity of 3500 mAh) and equipped with a charge controller (TP 4056 controller) for monitoring the voltage in order to protect the device against excessive charging or discharging. All components responsible for stethoscope operation were placed in the main body of the designed device in such a way to provide free access to replaceable elements and prioritize the requirement of a small size of the device.

### 2.2. Design of Stethoscope Housing

**Figure 1:**
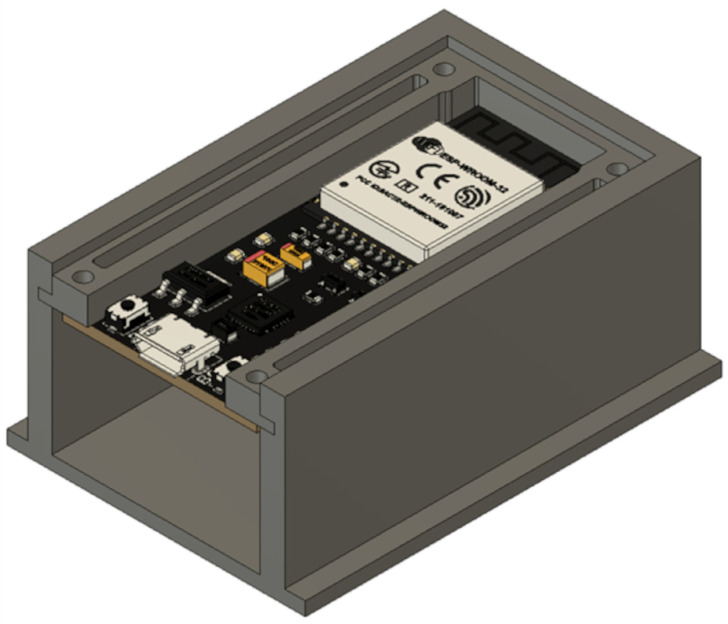
The CAD model of the fragment of the stethoscope housing with the main board fastening designed with Autodesk Inventor 2020.

The shape of the printed housing was adapted to the planned system of components. The first thing which was designed in the central part of the main body was the fastening of the main board (ESP32-38 Pines) controlling stethoscope operation (Figure 1).

In the pre-designing phase of the device housing, the following assumptions were defined: The device main body should have a modular form, as such a structure enables quick prototyping and the fast and comfortable assembling of the device and therefore contributes to faster implementation of the model in serial production. The housing should include a jack socket enabling the connection of headphones or loudspeakers, a switch, and a micro-USB port, which enables the communication with a computer. The structure of the housing must make it possible to replace the battery or to charge the storage battery. The model of the device housing was matched with previously selected electronic components, which have been presented above.

Taking into account the priority of device affordability, it was decided that the 3D printing method had to be used in the process of building the stethoscope main body. The above-mentioned method consists in the generation of three-dimensional physical objects on the basis of a computer model. Initially, it was used for the quick prototyping of elements; nowadays, it finds numerous applications in many fields. More and more attention has been paid to the application of this method in medicine, not only to the construction of diagnostic tools, as it is the case here, but also in the scope of clinical use, such as the creation of implants [15,16,17]. The popularity of 3D printing technology is on the rise proportionally to the current fall in its prices and increased availability [18,19,20]. Among a wide assortment of patented 3D printing methods, such as SLS (Selective Laser Sintering), LFS (Low-Force Stereolithography), PolyJet, MJF (Multi-Jet Fusion), HSS (High-Speed Sintering), DLMS (Direct Metal Laser Sintering), and SBJ (Sand Binder Jetting), the FDM technology (Fused Deposition Modelling) was selected. FDM consists in the fabrication of three-dimensional forms by means of the deposition of layers of polymers fed through a heated nozzle [21]. The FDM technology was chosen because the printed elements are durable and suitable for building functional prototypes. Additionally, a wide range of thermoplastic materials can be used. Disadvantages such as printing speed or the need for post-processing to obtain a smooth finish are not problematic in the case of our simple, small prototype [22,23].

### 2.3. Analysis of Materials Used for Construction of Stethoscope Main Body

Three-dimensional printing makes use of thermoplastics with different properties. The most popular materials applied in this method are as follows: ABS (acrylonitrile butadiene styrene copolymer), ASA (acrylonitrile styrene acrylate), PLA (poly(lactide)), PET-G (polyethylene terephthalate glycol copolymer), and nylon. The plastics (filaments) GRIP, MediFlex, PET-G, and PLA could be used in the designed device. It was taken into consideration that the properties of the filaments would be directly related to the assurance of the required strength parameters [19,20,24].

We planned to construct the housing of the device body from the material GRIP, belonging to a group of elastic TPU filaments (thermoplastic polyurethane elastomer), which is characterized by high dynamic friction and minimum shrinkage. It is often used for the manufacturing of soft-touch elements. This material is made from raw materials complying with directive EU 10/2011 concerning materials intended to come into contact with food and standard EN 71-3 related to the safety of toys [25,26].

The material MediFlex was also considered as an alternative, as it is characterized by high resistance to commonly used methods of sterilization, such as steam, EtO, and Gamma (25 kGy and 50 kGy); a wide range of working temperature of the polymer (from −50 °C to 125 °C); high mechanical strength; good chemical resistance—except for organic solvents and oils (with which the designed device will not come into contact); high resistance to weather conditions; and hardness 96 Shore A. The above-mentioned material is entirely produced from raw materials used for medical devices—such products satisfy the standards related to contact with food and the human body and even implantation.

Other elements of the device housing were designed to be made from the material PET-G, which is characterized by high chemical resistance, high strength, and great transparency. This material is produced from raw materials and dyes which are intended to come into contact with food, in compliance with directive EU 10/2011 [25]. These raw materials also meet the ISO 11607-1:2019 and ISO 10993 standards [27,28].

An alternative to PET-G is the material PLA (aliphatic polyester–polyactide), which is very popular in the field of manufacturing medical products due to its compatibility. Therefore, it is often used in stents, orthopaedic implants, tissue scaffolds, absorbable surgical sutures, dressings, and other hygienic products (such as napkins or diapers). PLA is characterized by high rigidity and small contraction, thanks to which it does not require a heated table or a heating chamber in 3D printing. The printing with this material is performed in relatively low temperatures—within the range from 190 to 220 °C. The material shows quite good tensile strength. Its advantage is that it is recyclable, while its drawback is that it has low resistance to temperature. However, in the case of the designed device, the latter is not so important, as the other electronic components of the device should not be subjected to intense heating either. The above-mentioned material is manufactured in accordance with directive EU 10/2011 [25].

A thorough analysis of the utility of the above-mentioned materials constituted an essential part of the project work, as we planned to make individual parts of the housing of the designed stethoscope from those materials. Taking into account the specification of particular materials, it was finally decided that the whole housing of the stethoscope was to be made of PLA. The main factors contributing to this choice were the affordability of the material, easy printing, and the properties enabling universal use of the product in medicine.

### 2.4. Strength Tests

Within the framework of pre-implementation investigations, static tensile tests were conducted on ten printed samples. In engineering practice, in the selection of a material for a certain purpose, a method of uniaxial tension testing is used. Conducting a tensile strength test on the printed samples of a printed stethoscope housing is crucial to assessing whether the material and 3D printing technology meet the requirements of durability, safety, and functionality of the medical device. The casing of the stethoscope is exposed to various loads during use, e.g., pressure, stretching during manipulation, or accidental falls. The tensile test ensures that the material will withstand everyday use. The 3D printing process can be performed with different fillings, which may cause defects such as microcracks, layer weakening (delamination) or porosity. The tensile test allows you to assess whether the printing parameters, such as the type of filling, temperature, or filling (percentages), ensure adequate strength. This type of examination is frequently applied due to the fact that such an experiment is easy to carry out and provides easy-to-calculate data on physical properties specific to a given material. This type of test is broadly used to determine material properties. The tests were performed in compliance with valid standards, PN-EN ISO 527-1:2020-01 and PN-EN ISO 527-2:2012 [29,30]. Profiles subjected to testing had a flat, ‘paddle-like’ form and the following dimensions: a thickness of 4.0 ± 0.2 mm, the width of the part subjected to measurements of 10 ± 0.2 mm, and a total length of more than 150 mm. Other important parameters are presented in Table 2.

The tensile test was carried out on ten specimens, which had been printed one by one by using the same 3D printer.

### 2.5. Construction of Stethoscope Housing

The main board (ESP32-38 Pines) was fixed by means of screws, enabling movable fastening. On the one hand, this allows for quick and easy mounting; on the other hand, it makes it possible to pull it out of the housing, for instance, in the case of replacement or modification of the soldered joints. In addition, a sound amplifier was also mounted onto the fastening of the main board.

**Figure 2:**
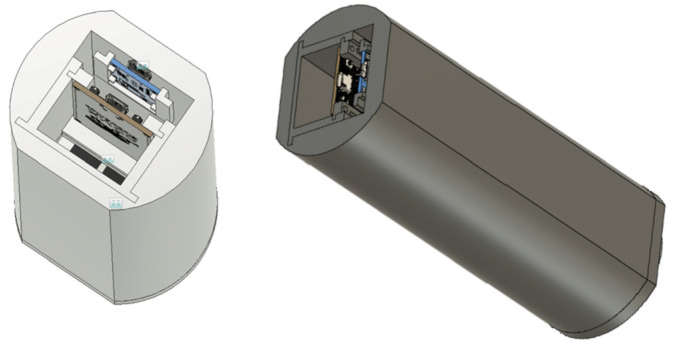
CAD model of stethoscope assembly designed with Autodesk Inventor 2020.

A basket with a Li-ion cell 18650 was stuck onto the lower part of the main body. A charging module was fixed to the upper wall of the housing by means of hot glue, directly under the module of the main board (Figure 2).

**Figure 3:**
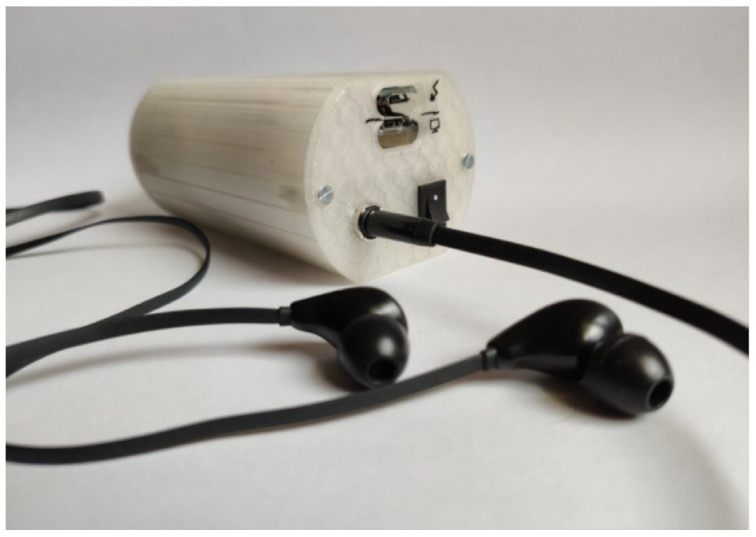
Electronic remote stethoscope (controlling side).

Such a position enables a micro-USB port to be located on the rear wall of the device main body. Next to the micro-USB port, on the rear wall, there is a diode, which signalizes the process of charging the battery and the end of the process. There is also a switch, and a jack fixed to the housing with a dedicated nut (Figure 3).

A microphone was fastened to the stethoscope chest piece by means of glue, which reinforced the sealing of the auscultation canal, improved the sound quality, and reduced external noise. The chest piece was mounted, also using glue, on the front wall of the device housing. Individual electronic elements were soldered together. Such a solution makes it possible to avoid failures related to the accidental disconnection or loosening of the components. The fact that soldered joints prevail over ‘gold pin’ joints contributes to the reduction in the device dimensions. The front and rear walls were fixed to the central part of the device main body with screws. Such a solution enables access to the internal structure of the device and enhances its durability. All elements of the main body were printed by using the printer Gembird FlashForge Guider 2S and the material PLA manufactured by Noctuo from Gliwice, Poland. The housing shown in Figure 3 was fastened by using screws and sealed with glue. Thanks to the application of the glue, a more solid construction was achieved, which meets the requirements in the scope of the device’s proper functioning.

## 3. Results

The designed housing of the stethoscope consists merely of four parts. The shape and structure of the interior of the main body are adapted to achieve the quick mounting of all necessary components. The housing structure also enables the modular assembly of the device at several workstations, which also speeds up the manufacturing process.

The minimization of the production costs of the stethoscope was achieved by the application of existing electronic components and 3D printing. The adopted FDM 3D printing technology enables quick and inexpensive prototyping. The reduction in manufacturing costs in this scope also results from the shortening of the time necessary to produce a given element [31].

The tensile test was carried out on ten PLA specimens, which had been printed one by one by using the same 3D printer (Gembird FlashForge Guider 2S).

The results are presented in Table 3.

On the basis of the conducted tests, it can be stated that the tensile strength of the sample with 50% hexagonal filling reaches almost 36 MPa. The elongation of the samples oscillates between 4 and 5%. Comparable values of the results from all 10 tests indicate that the investigations were carried out in a proper way. The PLA specification sheets issued by individual manufacturers assure that the tensile strength values of the material comply with the ASTM D882 standard [20] for injection samples (i.e., with 100% filling) at the level of 50–60 MPa (e.g., Finnotech: 53 MPa; Noctuo: 60 Mpa) [32,33,34]. At the same time, manufacturers warn that the declared values do not guarantee safety, and users must conduct all necessary tests and verify analyses themselves in order to confirm the safety compliance of the final products. However, such values constitute a point of reference, on the basis of which one can state that the obtained mean value of tensile strength in the case of samples filled up to 50% amounting to 35.89 MPa is satisfactory and sufficient. Therefore, it is a clear indication that such a material can be used in the implementation of the designed stethoscope.

It is worth comparing the above-mentioned values with the results of tests carried out on samples with 100% filling presented by Miazio, which reveal that in the case of the sample with complete filling, the tensile strength σ_M_ amounted to 53 MPa [35]. This result is comparable with the results obtained in the tests performed by Raja et al., where the values from five tests were 47.55, 50.23, 48.56, 49.02, and 47.95 MPa, producing the mean value of 48.66 MPa [36].

Taking into consideration the fact that the samples which were to be used in tensile strength tests were printed with 50% filling, the obtained mean result at a level of 36 MPa should be deemed satisfactory in comparison with the tests performed on samples with 100% filling. The value of strength depends to a considerable degree on the type and degree of filling and substantially increases above 70%. The mechanical properties of the material do not constitute an obstacle to its application in the production of the main body of the designed stethoscope.

**Figure 4:**
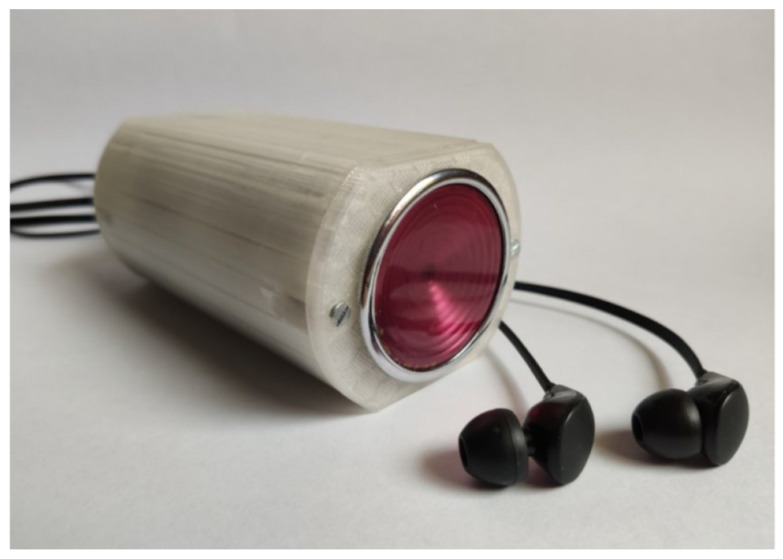
Electronic remote stethoscope (auscultation side).

The designed and manufactured housing meets the requirements which were initially assumed. The device has a small size (5 × 6 × 10.7 cm) and weighs 206 g. The housing ensures proper position and distribution of the individual elements inside the device. This enables comfortable use and possible modifications of the device. The stethoscope can be placed in the pocket of a doctor’s coat. The stethoscope can be used in a similar way to a traditional device, but the auscultatory sounds are transmitted through headphones (Figure 4). The doctor can choose headphones tailored to their individual needs and preferences.

The stethoscope features charging inputs, a switch, and communication inputs, such as headphones, external loudspeakers, and a micro-USB port enabling communication with the computer.

**Figure 5:**
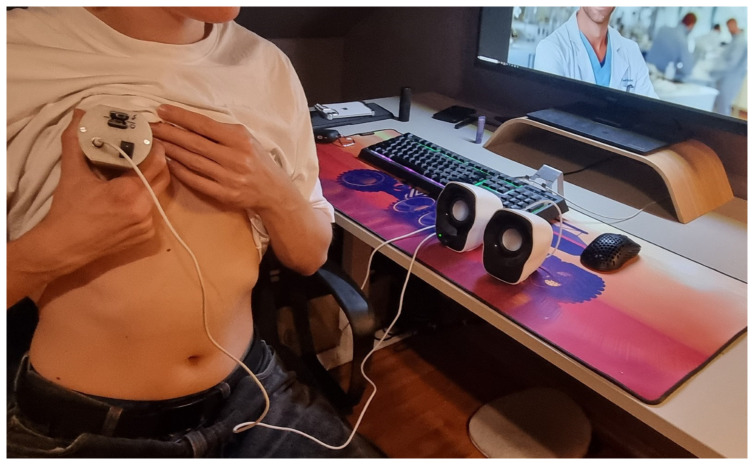
Tele-advice.

The stethoscope enables the conduction of remote examinations, facilitating auscultation from a distance, particularly in situations where in-person consultations are not feasible (Figure 5).

The stethoscope is relatively straightforward to use; however, improper placement of the chest piece equipped with a diaphragm on the body, excessive or insufficient pressure, or selecting an incorrect auscultation site can lead to inaccurate results. Nevertheless, this procedure can be conducted under the supervision of a physician who, via a camera, can observe the process and provide guidance on how to position the device correctly (Figure 5).

**Figure 6:**
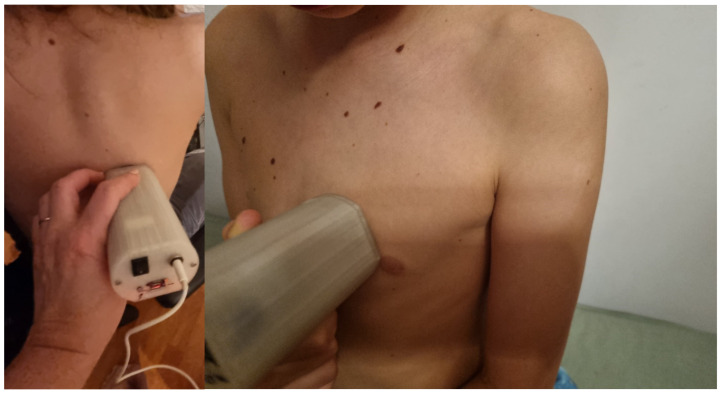
An examination using a stethoscope.

The examination can be performed at any time, allowing for frequent assessments as needed (Figure 6).

**Figure 7:**
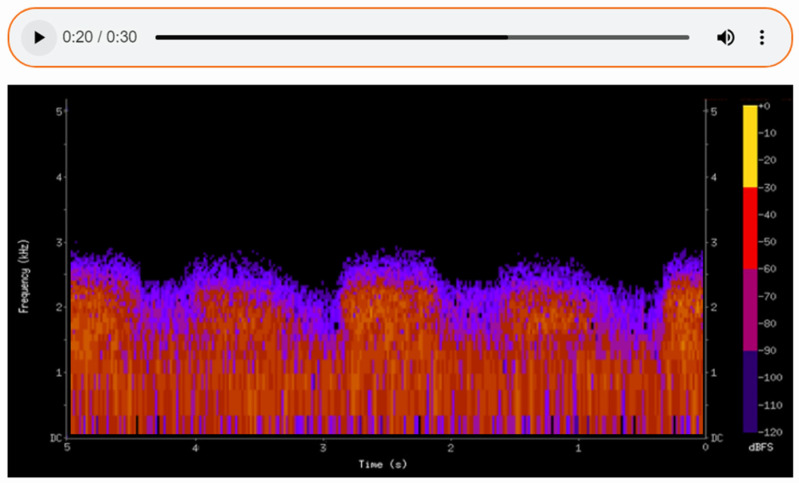
The spectrogram of a healthy lung.

**Figure 8:**
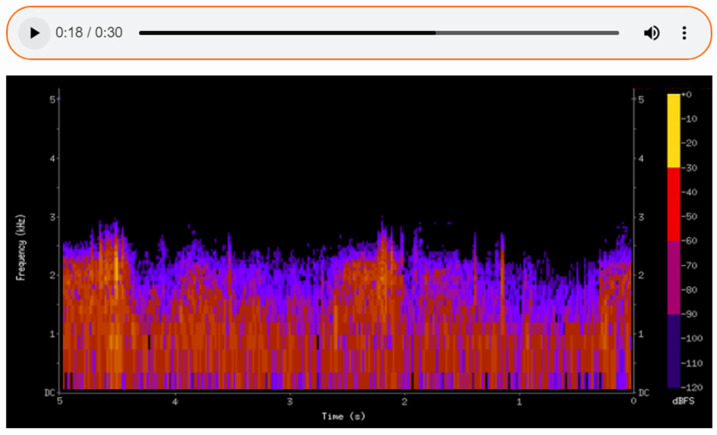
A spectrogram showing pathological changes during the auscultation of the lungs.

**Figure 9:**
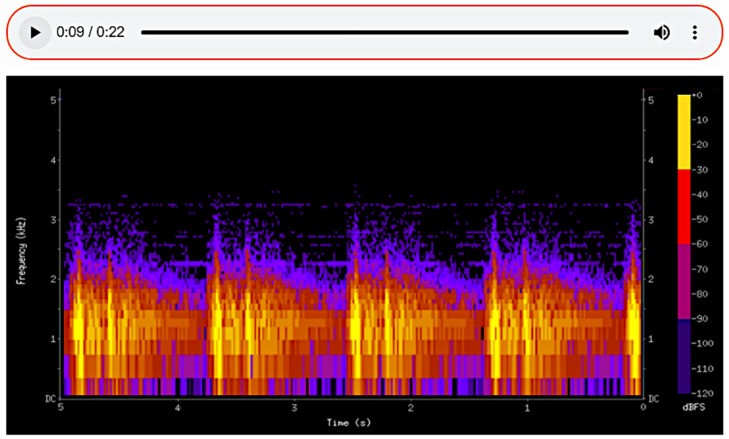
The spectrogram of the heartbeat.

The sounds detected during auscultation can be recorded, enabling detailed subsequent analysis and facilitating consultation with other specialists. By translating auditory data into a visual format, spectrograms (Figure 7, Figure 8 and Figure 9) allow physicians to make more informed decisions and contribute to improved patient outcomes in care. Additionally, the ability to visualize the sound waveforms aids in more precise interpretation (Figure 7, Figure 8 and Figure 9).

The physician can review the recorded audio later, allowing for thorough interpretation and, if necessary, consultation with a second specialist. These recordings can also be stored in the patient’s medical records, enabling longitudinal comparisons and the tracking of changes over time. Changes in sound patterns over time can be tracked, providing valuable insights into disease progression or the effectiveness of treatments.

In the first spectrogram (Figure 7), we observe normal vesicular breath sounds. This type of sound is associated with healthy lung function, where air flows freely through the airways into the alveoli. The even and consistent patterns of the spectrogram confirm the absence of any pathological changes in the respiratory activity. On the spectrogram of healthy lungs, it is possible to distinguish between the inhalation and exhalation phases of breathing. These phases are clearly visible as separate segments of the sound pattern, with inhalation typically appearing as a smoother, more continuous sound and exhalation being slightly shorter and less intense. The spectrogram demonstrates a rhythmic and regular pattern, reflecting the natural, cyclical nature of normal breathing. This rhythmicity and the clear distinction between phases are key indicators of healthy lung function, confirming the absence of abnormalities in airflow.

The second spectrogram depicts fine crackles, which are discontinuous sounds often associated with pathological conditions such as pneumonia.

The recorded audio can be reviewed by the physician at a later time, allowing for thorough interpretation and, if necessary, consultation with a second specialist.

The spectrogram of the heartbeat shows a normal result. The sound waves exhibit a distinct and regular rhythm, typical of healthy heart function, which verifies the device’s accuracy and correct performance. (Figure 9)

The spectrogram allows clinicians to detect specific frequency patterns that correspond to different pathological conditions. The spectrogram provides quantifiable data, such as the duration, frequency range, and intensity of lung or heart sounds. This helps to standardize diagnoses and reduce variability among practitioners. Spectrograms offer a visual representation of sounds, making them a tool for tutoring medical students and trainees. They help bridge the gap between theoretical knowledge and practical auscultation skills.

## 4. Discussion

The above-described stethoscope, executed within the framework of this project, was checked by a physician with a medical licence. The device features high sensitivity, which is a condition for making a good diagnosis. By using a jack, the device may be connected to loudspeakers or headphones, which allows for the adjustment of the sound volume. It is also possible to improve the sound quality by increasing the frequency of sampling, e.g., to studio sound quality (44.1 kHz), by mounting a more expensive microphone model.

Each visit to a care institution (hospital or health clinic) is a consequence of the patient’s discomfort and stress. More and more attention is paid to the impact of home treatment on the psyche and faster recovery of patients [37,38,39]. Home convalescence often involves the patient using easy-to-use medical tools. Therefore, the use of the stethoscope presented in the article, which could reduce visits to health centres, seems to be a valuable solution. The use of medical instruments at home and their operation by patients themselves is becoming an increasingly common and desirable phenomenon (in addition to the factors mentioned above, also due to the age structure of society). The organisation of remote technical assistance (personal and virtual) is related to their service [40,41].

There are many electronic stethoscopes that can be improved. It is important that they can be introduced into wide production. It is essential that the product implemented on the market is easy to use for the people who are to use it. This can improve diagnostics through accuracy and the ability to record and compare sounds [4]. Research is also being conducted into the possibility of using higher-quality microphones [42].

## 5. Conclusions

As a result of the presented process of designing and manufacturing an electronic stethoscope, we created a device designed for the remote diagnosis of lung and heart diseases. In its construction, we used existing electronic components, a classical single-sided chest piece, an on/off switch, a charging input, communication ports, and sockets to enable the connection and communication of speakers and headphones with a computer. The modular designed and manufactured form of the body in which they are housed ensures the rapid prototyping and quick and convenient assembly of the device. Strength tests carried out on PLA, static tensile tests, confirmed the suitability of this material for the intended purpose. The design of the housing, the layout of the individual components of the device, the way in which they are attached and joined together, ensures user comfort. The communication ports provide various possibilities for using the device both for direct examination (by the doctor) and for remote consultation. Another important advantage provided by the device is the ability to record the sounds detected during the examination, which allows for detailed analysis at a later date and facilitates consultation with other specialists. In addition, the recorded audio data can be converted into a visual format (spectrogram), as presented in the article. The visualization of the audio waveforms is helpful for a more precise interpretation of the auscultation results. The main achievements of the work include the design of a stethoscope with empirically proven high sensitivity, capable of accurately capturing sounds and providing the ability to connect to external devices such as loudspeakers or headphones to adjust volume and improve sound quality. The designed stethoscope fulfils the adopted initial design assumptions placing among the priorities affordable production costs, ease of use of the device by nonmedical people, and provision of data transfer capabilities. The design of the device responds to the increasing need for and popularity of remote medical consultations. To improve device design, a wireless data flow and automatic data recording and storing may be used. However, the main objective of this project was to construct a stethoscope device enabling the auscultation of the patient during a medical online consultation, which has been achieved. The affordability of the device was one of the chief factors taken into consideration, hence the priority of a maximum reduction in manufacturing costs. The possibility of remote auscultation of the patient in the diagnostic process greatly facilitates the physician’s work and is a comfortable option for the patient. The stethoscope enables the remote monitoring of health conditions, facilitating the non-invasive diagnosis and accurate detection of vital functions and the rapid detection of abnormalities.

## Figures and Tables

**Table 1:** Advantages and disadvantages of a traditional stethoscope.

Advantages	Disadvantages
Traditional (doctors accustomed to using it)	Cannot record or share audio
Lightweight	Uncomfortable (headset = metal part of the stethoscope can be uncomfortable; ear tips are soft, but can cause discomfort or irritation)
Inexpensive	Requires proper technique to avoid user errors
No dependence on batteries or power	Only a professional can detect the disease
	Unsuitable for hearing-impaired practitioners

**Table 2:** The printing parameters used.

Filling	50%
Type of filling	Hexagonal
Table temperature	50 degrees Celsius
Head temperature	205 degrees Celsius

**Table 3:** Results of tensile tests conducted on 10 samples printed with FDM method by using PLA filament.

Test	σ_x1_	σ_M_	ε_M_	σ_B_	ε_B_	h	b	A_0_
	MPa	MPa	%	MPa	%	mm	mm	mm^2^
Test 1	34.65	36.01	3.75	21.64	5.62	4.11	10.36	42.08
Test 2	33.24	34.54	3.47	20.98	4.65	4.15	10.14	42.08
Test 3	34.29	36.25	3.53	21.05	5.08	4.12	10.23	42.14
Test 4	32.028	35.95	3.70	20.08	4.92	4.15	10.26	42.58
Test 5	32.52	35.81	3.49	21.00	4.46	4.13	10.24	42.29
Test 6	31.17	36.59	3.53	22.36	5.44	4.14	10.16	42.06
Test 7	28.66	35.81	3.47	21.18	4.68	4.18	10.22	42.71
Test 8	29.18	35.89	3.42	20.80	4.83	4.11	10.19	41.88
Test 9	32.28	35.78	3.48	20.48	4.50	4.12	10.26	42.27
Test 10	28.97	36.21	3.36	22.06	4.39	4.11	10.26	42.16
Mean value	31.70	35.89	3.52	21.16	4.86	4.13	10.23	42.23

Above, the following apply: σ_x1_—stress at elongation x1%. σ_M_—tensile strength. ε_M_—relative elongation at maximum tensile stress. σ_B_—stress at rupture. ε_B_—total elongation/relative elongation at rupture. h—thickness of ‘paddle-like’ sample. b—width of part subjected to measurements. A_0_—surface of initial cross-section of measurement sample segment.

## Data Availability

Data are contained within the article.
